# Association between macrocalcification and papillary thyroid carcinoma and corresponding valuable diagnostic tool: retrospective study

**DOI:** 10.1186/s12957-023-03016-7

**Published:** 2023-05-16

**Authors:** Mengyao Ye, Shan Wu, Qi Zhou, Fang Wang, Xiaojun Chen, Xiaohua Gong, Wenjun Wu

**Affiliations:** 1 Department of Endocrinology, Wenzhou Hospital of Integrated Traditional Chinese and Western Medicine, Wenzhou, Zhejiang 325015 China; 2 Department of Endocrinology, First Affiliated Hospital of Wenzhou Medical University, Wenzhou, Zhejiang 325015 China; 3 Department of Endocrinology, People’s Hospital of Yuhuan, Taizhou, Zhejiang 318000 China; 4 Departments of Pathology, First Affiliated Hospital of Wenzhou Medical University, Wenzhou, Zhejiang 325015 China

**Keywords:** Thyroid nodule, Papillary thyroid carcinoma, Macrocalcification, Fine needle aspiration biopsy, Proto-Oncogene Proteins B-raf V600E

## Abstract

**Background:**

Microcalcifications are suggested to be an indicator of thyroid malignancy, especially for papillary thyroid carcinoma (PTC), nonetheless, the association between macrocalcification and PTC is underexplored. Furthermore, screening methods like ultrasonography and ultrasound-guided fine needle aspiration biopsy (US-FNAB) are limited in evaluating macro-calcified thyroid nodules. Thus, we aimed to investigate the relationship between macrocalcification and PTC. We also explored the diagnostic efficiency of US-FNAB and proto-Oncogene Proteins B-raf V600E (BRAF V600E) mutation in macro-calcified thyroid nodules evaluation.

**Methods:**

A retrospective research of 2645 thyroid nodules from 2078 participants was performed and divided into three groups as non-, micro-, and macro-calcified for further PTC incidence comparison. Besides, a total of 100 macro-calcified thyroid nodules with both results of US-FNAB and BRAF V600E mutation were screened out for subsequent evaluation of diagnostic efficiency.

**Results:**

Compared to non-calcification, macrocalcification showed a significantly higher incidence of PTC (31.5% vs. 23.2%, *P*<0.05). Additionally, when compared with a single US-FNAB, the combination of US-FNAB and BRAF V600E mutation showed better diagnostic efficiency in diagnosing macro-calcified thyroid nodule (area under the curve (AUC) 0.94 vs. 0.84, *P*=0.03), with a significantly higher sensitivity (100.0% vs. 67.2%, *P*<0.01) and a comparable standard of specificity (88.9% vs. 100.0%, *P*=0.13).

**Conclusions:**

Occurrence of macrocalcification in thyroid nodules may suggest a high risk of PTC, and the combination of US-FNAB and BRAF V600E showed a greater value in identifying macro-calcified thyroid nodules, especially with significantly higher sensitivity.

**Trial registration:**

The Ethics Committee of The First Affiliated Hospital of Wenzhou Medical University (2018-026).

**Supplementary Information:**

The online version contains supplementary material available at 10.1186/s12957-023-03016-7.

## Background

Microcalcification is suggested to be linked with papillary thyroid carcinoma (PTC) [1, 2]; however, the relationship between macrocalcification and PTC remains inconclusive; some reports suggested that macro calcification was also a risk factor for thyroid malignancy [3, 4], while some showed otherwise [5, 6]. Furthermore, current imaging modalities have limitations in estimating macrocalcifications. Ultrasonography assessment is suggested as the preferred non-invasive screening tool of thyroid nodules in regular clinical practice; however, it would be severely restricted by calcification when depicturing ultrasound features like shape, margin as well as size, and thus cause a low efficacy of ultrasonographic-based thyroid imaging reporting and data system in evaluating macro-calcified nodules. Ultrasound-guided fine needle aspiration biopsy (US-FNAB), is suggested as a reliable and cost-effective approach for differentiating malignant from benign thyroid nodules [4, 7]. Besides compared to the ultrasonography, US-FNAB has a higher resolution which may help in identifying undefined nodules (Bethesda category I/III/IV). While proto-Oncogene Proteins B-raf V600E (BRAF V600E) mutation, a dependable molecular marker of PTC, is suggested as a reliable auxiliary tool of US-FNAB in evaluating macro-calcified nodules, it has been reported that the use of both US-FNAB and BRAF V600E mutation increased sensitivity as well as specificity for PTC diagnosis, and significantly reduced corresponding false-negative rate [8]. However, studies relating its predicative value of macro-calcified nodules are underexplored.

Our current study aimed to investigate the relationship between macrocalcification and PTC in thyroid nodules, and further explore the diagnostic efficiency of US-FNAB and BRAF V600E mutation in macro-calcified thyroid nodules evaluation.

## Methods

This was a retrospective study approved by the Ethics Committee of The First Affiliated Hospital of Wenzhou Medical University (2018-026) and was conducted with a waiver of patient informed consent. The study followed the Declaration of Helsinki.

### Subjects and study design

**Fig. 1:**
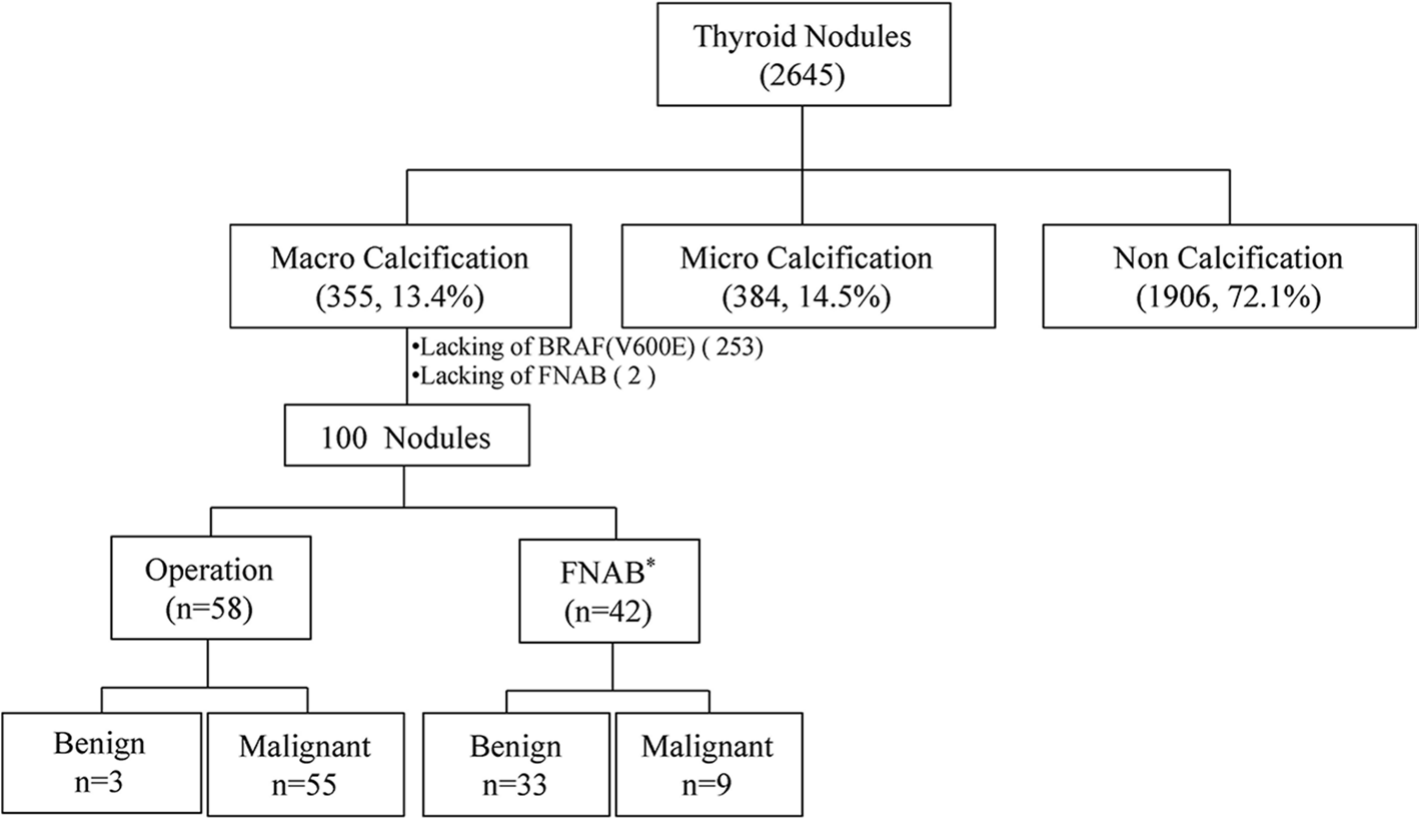
Flow chart

We retrospectively included a total of 2645 thyroid nodules from 2078 participants, who were diagnosed with nodular thyroid disease in the first affiliated hospital of Wenzhou Medical University between February 2016 and August 2017 (Fig. 1). Participants’ medical records were reviewed up to June 2020 to gather relevant data. Inclusion criteria included (a) thyroid nodules were confirmed on histologic examination of surgical specimens or core needle biopsy (CNB) and (b) nodules that were not histologically confirmed were subjected to US-FNAB at least twice and follow-up ultrasound examinations for at least 2 years. Results of surgical resection specimen or cytological specimen from CNB (for non-surgical thyroid nodules only) were taken as gold standard for nodule pathological diagnosis; otherwise, the most recent cytological results of US-FNAB were taken as gold standard if the patient received neither operation nor CNB. And based on our previous research, the malignant rate of nodules in Bethesda V and VI was more than 97.2% in our hospital, thus in this study, we ascribed Bethesda V~VI as malignancy, while Bethesda I~IV as benign [9]. Specimens with other types of thyroid cancer like follicular carcinoma, medullary carcinoma, and undifferentiated carcinoma of the thyroid were all excluded, and only PTC was included. Each subject could have more than one nodule. All the nodules were classified into three groups according to the maximum diameter of calcification in ultrasound as no calcification, microcalcification (<2 mm), and macrocalcification group (≥2 mm) [1]. In addition, we screened out a total of 100 macro-calcified thyroid nodules, which possessed both results of US-FNAB and BRAF V600E, for further evaluation of diagnostic efficiency.

### US-FNAB and BRAF V600E mutation

The method has been well detailed in a previous study [10]. Briefly, color Doppler ultrasound (EZU-MT28-S1; Hitachi Medical, Tokyo, Japan; 5~12 MHz linear probe) was used for US-FNAB. Each lesion was aspirated by 23-gauge needles attached to a 2-ML syringe for 2~3 passes in different directions. The aspirates were expelled and smeared into glass, then immediately placed in 95% alcohol for fixation. Then the samples were sent for cytologic analysis by two experienced cytologists and the reports of cytology were classified based on the Bethesda thyroid reporting system (category I: undiagnosed or unsatisfactory specimen; II: benign lesion; III: unclear cellular atypical lesion or unclear follicular lesion; IV: follicular lesion or suspicious follicular tumor; V: suspicious malignant tumor; VI: malignant tumor) [11]. The material remaining from the specimen after cytological preparation was collected in 180-µL cytolysis liquid and was stored at −20°C for subsequent for BRAF V600E mutation analysis. Amplification refractory mutation system was used to detect BRAF V600E mutation with commercial kit (ADx-Amplification Refractory Mutation System [ADx-ARMS]; AmoyDX, Xiamen, China).

### Statistical analysis

Continuous variables were shown as means (standard deviation, SD) while categorical variables were shown as percentages (%). Data with normal distribution and homogenous variance was statistically tested by using independent *t* test or one-way ANOVA; Mann–Whitney *U* test or Kruskal-Wallis test where appropriate. Chi-square test or Fisher’s exact test was used for statistical analysis of the classifying variables. Bonferroni was used for post hoc tests. The area under the receiver operating characteristic (AUROC) was used to determine the diagnostic accuracy of the analyzed parameters discriminating the data from different imaging modalities. *Z* statistic was calculated by the formula *Z*=(AUC1—AUC2)/√(SE1^2^ +SE2^2^). SPSS (23.0) were used for statistical analysis in this part. *P*-value less than 0.05 (two-tailed) was considered significant for all analyses.

## Results

### Basic characteristics of participants

We enrolled two thousand six-hundred and forty-five (2645) participants in our study; out of the 2645, 355 had macrocalcification, 384 had microcalcification while 1906 had non-calcification. There were no significant differences among three groups when gender was compared (*P*=0.18). There were significant differences in the age (*P*<0.01) and The Bethesda System for Reporting Thyroid Cytopathology (*P* <0.01) among the three groups. Table 1 showed the enrolled participants’ information.

**Table 1:** Basic characteristics

	Macrocalcification (355)	Microcalcification (384)	Non-calcification (1906)	*P* value
Gender (male, %)	68 (19.2%)	94 (24.5%)	399 (20.9)	0.18
Age (mean ± SE)	51.4±0.23^a^	46.0±0.60^b^	47.5±0.27^b^	<0.01^*^
TBSRTC				<0.01
Unreported	2 (0.6%)^a^	1 (0.3%)^a^	13 (0.7%)^a^	
I/III/IV	53 (14.9%)^a^	33 (8.6%)^b^	130 (6.8%)^b^	
II/V/VI	300 (84.5%)^a^	350 (91.1%)^b^	1763 (92.5%)^b^	

Unless additionally described, values were presented as number, %

*Abbreviations*: *SE* Standard error, *TBSRTC* The Bethesda System for Reporting Thyroid Cytopathology

^*^One-way ANOVA, otherwise chi-square test was used

Values labeled without a common superscript letter differ from each other in the same row, *P* <0.05

### PTC incidence among non-calcified nodules, micro-calcified nodules, and macro-calcified nodules groups

The comparison of PTC incidence among the three groups was summarized in Table 2. The PTC incidence of macro and microcalcification nodules were both significantly higher than that in non-calcified nodules (53.9% vs 23.2%, *P*<0.05; 31.5% vs 23.2%, *P*<0.05), though micro and macro-calcified groups also showed significant difference (53.9% vs 31.5%, *P*<0.05). Since the mean age of macrocalcification was significantly older, we also did further stratified analysis by age. Data showed that the above result of significant difference in PTC incidence among the three groups kept stable in both people younger than 50 years old (61.8% vs. 38.0% vs. 26.7%, *P*<0.01) and those who were older than 50 years old (43.3% vs. 26.8% vs. 18.8%, *P*<0.01). Besides, our data also showed that, for people younger than 50 years old, the PTC incidence was significantly higher than those who were older in macrocalcification (38.0% vs. 26.8%, *P*=0.03), microcalcification(61.8% vs. 43.3%, *P*<0.01) as well as non-calcification (26.7% vs. 18.8%, *P*<0.01).

**Table 2:** Comparison of PTC incidence among groups and corresponding stratified analysis by age

	Macrocalcification	Microcalcification	Non-calcification	*P* Value
PTC	112 (31.5%)^a^	207 (53.9%)^b^	443 (23.2%)^c^	<0.01
Age stratified				
<50 years	57/150 (38.0%)^a^	136/220 (61.8%)^b^	284/1062 (26.7%)^c^	<0.01
≥50 years	55/205 (26.8%)^a^	71/164 (43.3%)^b^	159/844 (18.8%)^c^	<0.01
*P* value	0.03	<0.01	<0.01	

Values were presented as number, %

Chi-square test was used and Bonferroni was used for post hoc tests

*Abbreviations*: *PTC* Papillary thyroid carcinoma

Values labeled without a common superscript letter differ from each other in the same row, *P* <0.05

### Diagnostic accuracy of combined US-FNAB with BRAF V600E mutation in macro-calcified nodules

**Fig. 2:**
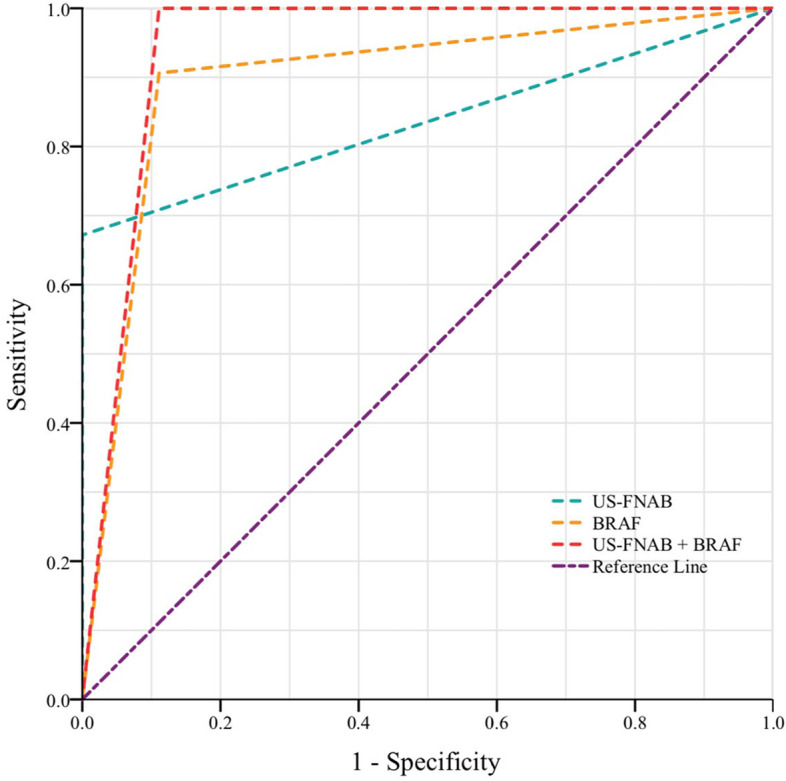
ROC curve

Totally, 100 macro-calcified nodules, which contain both outcomes of Bethesda classification and BRAF V600E, were included in this part. Our data showed that, compared to US-FNAB, the area under the curve (AUC) of US-FNAB combined BRAF V600E mutation was significantly improved (0.94 vs 0.84, *P*=0.03, Fig. 2), with a significantly higher diagnostic accuracy (96.0% vs 80.0%, *P*<0.01) and sensitivity (100.0% vs 68.2%, *P*<0.01), whereas the specificity showed no significant difference between groups (100.0% vs. 94.1% *P*=0.13) (Table 3).

**Table 3:** Comparison of malignancy-differentiating ability between FNAB and FNAB & BRAF among nodules with macrocalcification

	FNAB	FNAB and BRAF V600E	*P* value
Area under the curve	0.84	0.94	0.03^‡^
Sensitivity (%)	67.2%	100.0%	<0.01
Specificity (%)	100.0%	88.9%	0.125
Diagnostic accuracy	80.0%	96.0%	<0.01^†^

Corresponding fourfold table and calculation formula can be found in Supplementary Tables 1 and 2

*Abbreviations*: *FNAB* Fine needle aspiration biopsy, *BRAF V600E* Proto-Oncogene Proteins B-raf V600E

†Chi-square test, ‡Z test, otherwise McNemar’s test with exact significance was used

## Discussion

Our study demonstrated that though microcalcification might be more specific for PTC, macrocalcification was also tightly associated with PTC. In addition, compared to US-FNAB, combined US-FNAB with BRAF V600E mutation has obviously superior PTC differentiating ability for macro-calcified thyroid nodules, especially with a significantly higher sensitivity.

Calcification, especially microcalcification, can be seen in both benign and malignant thyroid nodules, however, it has been reported to be more common in malignancy [12, 13]. Most of the current studies have revealed a critical role of microcalcification in suggesting PTC [1, 2], while the clinical significance of macrocalcification was under debate [3–5]. Consistent with some of the previous studies, our data showed a significant increase of PTC incidence in both two calcified groups when compared to the non-calcified group. However, an even higher incidence of PTC was shown in micro-calcified thyroid nodules. This result suggested that calcification was one of the critical signs of PTC, but microcalcification could be a better predictor [1, 3]. Notwithstanding, we still consider macrocalcification being worthy of attention, because same as our results (a malignancy of 31.5% in macro-calcified group), some studies also indicated a considerable portion of the malignancy in macro-calcified nodules, as Kim, et al reported a malignancy of 66% (116/174) in macro-calcified thyroid nodules, and Taki’s group reporting 49% (21/43) [14, 15].

One of the possible reasons for the complex clinical significance of macrocalcification was that the relationship between malignancy and macrocalcifications varied from its diverse ultrasonic morphologic characteristics. Park, et al reported that macrocalcifications with interruption or irregular thickness presented a significantly higher risk of malignancy [4]. And Lu Yin et al. reported a similar result that eggshell discontinuous macrocalcifications and irregular macrocalcifications occurred mainly in PTC [16]. Regrettably, the rough classification, with thyroid nodules being merely divided into micro-, macro-, and non-calcified, failed us further verifying this conjecture in this study.

Another potential influence factor might be age. Zhihong Wang et al. and Ning Wang et al. have reported that the relative risk of malignancy incidence was significantly higher in those younger than 45 years old with calcification or microcalcification [17, 18]. A similar result was also seen in our study that the risk of malignancy incidence was significantly higher in those younger than 50 years old in the macro-calcified group (38.0% vs 26.8%, *P*=0.03) and micro-calcified group (61.8% vs 43.3%, *P*<0.01). Nevertheless, whether the presence of macrocalcification in young patients predicted a higher risk of PTC remains to be further studied, since a similar significant difference was also seen in the non-calcified group in this study (26.7% vs 18.8%, *P*<0.01). Studies with larger sample sizes and a comparably balanced age distribution might be helpful in excluding the influence of selection bias.

Simultaneously, we also probed into whether US-FNAB combined BRAF V600E mutation had significantly stronger PTC differentiating ability in macrocalcifications. US-FNAB was currently the most useful and important nonsurgical approach for thyroid malignancy screening. Nonetheless, macrocalcification has posed a dilemma for US-FNAB in characterizing thyroid nodules. On the one hand, as we depicted above, macrocalcification per se should be classified as a “high-risk” sign for PTC and thus highly demand US-FNAB for diagnostic screening. However, the macrocalcification, on the other hand, limited the diagnostic value of US-FNAB due to a significantly higher incidence of inadequate sampling and non-diagnostic cytology (I/III/IV) [7]. While US-FNAB combined BRAF V600E mutation seemed to be a good way out. BRAF V600E has been wildly accepted to be strongly associated with the development as well as the progression of PTC and was thus recognized as a highly effective risk-predicting molecular marker for PTC [19–21]. It was almost absent in benign thyroid nodules and performed to be highly specific for PTC [22]. Furthermore, the current development of the amplification technique has enabled trace samples being easily sufficient and qualified with the detective requirements. And since macrocalcification often caused unsatisfied sampling, this progress may help BRAF V600E well complement with US-FNAB in evaluating macro-calcified thyroid nodules. Our research certificated such an advantage. Our data indicated that, compared to a single US-FNAB, its combination with BRAF V600E mutation performed a significantly higher accuracy and sensitivity, whereas a comparable specificity in predicting PTC among macro-calcified thyroid nodules. Similar results have also been reported previously in some researches involving thyroid nodules (not limited to macro-calcified nodules) [8, 21, 23]. However, one of the disadvantages of our study was that we did not compare the diagnostic value of US-FNAB combined BRAF V600E to CNB, which was another screening test with both high sensitivity and specificity, because some of the recent reports indicated that CNB showed greater diagnostic performance and ought to be considered first to improve diagnostic efficiency when evaluating entirely calcified types of macro-calcified nodules [24, 25]. Furthermore, the cost of the union test was higher than CNB. Therefore, although the union test was more secure and minimally invasive, further studies are needed to compare the cost-efficacy and diagnostic accuracy of these two methods in macro-calcified thyroid nodules. Last but not least, the number of macro-calcified thyroid nodules of 100 was small, additional studies with larger sample sizes are required to confirm current findings.

## Conclusion

In a word, our study demonstrated that macrocalcification was also an important malignant ultrasound sign for PTC. Combination of US-FNAB and BRAF V600E could improve the diagnostic accuracy of macro-calcified thyroid nodules, especially with a significantly higher sensitivity, whereas studies with a more detailed classification of macrocalcification as well as a larger sample size are needed for further testifying.

## Supplementary Information

**Additional file 1: Supplementary Table 1.** Fourfold table of macro-calcified nodules classification by using US-FNAB cytological diagnosis. **Supplementary Table 2.** Fourfold table of macro-calcified nodules classification by combined US-FNAB cytological diagnosis with BRAF(V600E). 

## Data Availability

The datasets generated and analyzed during the current study are available from the corresponding author on reasonable request.

## Abbreviations

PTC: Papillary thyroid carcinoma
US-FNAB: Ultrasound-guided fine needle aspiration biopsy
BRAF V600E: Proto-Oncogene Proteins B-raf V600E
CNB: Core needle biopsy
SE: Standard error
ROC: Receiver operating characteristic
AUC: Area under the curve
SEN: Sensitivity
SPE: Specificity
PPV: Positive predictive value
NPV: Negative predictive value
