# Supplementary material for: Association between macrocalcification and papillary thyroid carcinoma and corresponding valuable diagnostic tool: retrospective study
Source: World J Surg Oncol. 2023 May 16;21:149. doi: 10.1186/s12957-023-03016-7 (PMC10186772; doi:10.1186/s12957-023-03016-7)
Supplement: Supplementary file 1 — Additional file 1: Supplementary Table 1. Fourfold table of macro-calcified nodules classification by using US-FNAB cytological diagnosis. Supplementary Table 2. Fourfold table of macro-calcified nodules classification by combined US-FNAB cytological diagnosis with BRAF(V600E). [file 12957_2023_3016_MOESM1_ESM.docx]

Supplementary Table 1. Fourfold table of macro calcified nodules classification by using US-FNAB cytological diagnosis.

|  | | US-FNAB | |
| --- | --- | --- | --- |
|  |  | Malignancy | Benign |
| Gold Standard | Malignancy | 43 | 21 |
|  | Benign | 0 | 36 |

Bethesda I~IV were regarded as benign while Bethesda V~VI were regarded as malignancy in TBSRTC.

Sensitivity = TP/(TP + FN) × 100%

Specificity = TN/(TN + FP) × 100%

Positive predictive value (PPV) = TP/(TP + FP) × 100%

Negative predictive value = TN/(TN + FN) × 100%

Accuracy = (TP + TN)/(TP + TN + FP + FN) × 100%

Abbreviation: TBSRTC, the Bethesda System for Reporting Thyroid Cytopathology; TP, true positive; TN, true negative; FP, false positive, FN, false negative.

Supplementary Table 2. Fourfold table of macro calcified nodules classification by combined US-FNAB cytological diagnosis with BRAF(V600E).

|  | | US-FNAB & BRAF(V600E) | |
| --- | --- | --- | --- |
|  |  | Malignancy | Benign |
| Gold Standard | Malignancy | 64 | 0 |
|  | Benign | 4 | 32 |

Nodules classified as Bethesda I~~IV with negative BRAF(V600E) were regarded as benign, otherwise malignancy.

Sensitivity = TP/(TP + FN) × 100%

Specificity = TN/(TN + FP) × 100%

Positive predictive value (PPV) = TP/(TP + FP) × 100%

Negative predictive value = TN/(TN + FN) × 100%

Accuracy = (TP + TN)/(TP + TN + FP + FN) × 100%

Abbreviation: TBSRTC, The Bethesda System for Reporting Thyroid Cytopathology; BRAF, V-Raf murine sarcoma viral oncogene homolog B1; TP, true positive; TN, true negative; FP, false positive, FN, false negative.
